# Validity and reliability of the Turkish version of the topical corticosteroid phobia (TOPICOP) scale

**DOI:** 10.55730/1300-0144.6109

**Published:** 2025-11-04

**Authors:** Esra AĞAOĞLU, Hilal KAYA ERDOĞAN, Hilal ÇAVUŞ, Selma METİNTAŞ

**Affiliations:** 1 Department of Dermatology, Faculty of Medicine, Eskişehir Osmangazi University, Eskişehir, Turkiye; 2 Department of Public Health, Faculty of Medicine, Eskişehir Osmangazi University, Eskişehir, Turkiye

**Keywords:** TOPICOP scale, Turkish, validity, reliability

## Abstract

**Background/aim:**

Concerns about the use of topical corticosteroids (TCSs), commonly referred to as corticophobia, are widespread among dermatology patients and often lead to nonadherence to TCS treatment. This study aimed to evaluate the validity and reliability of the Turkish version of the topical corticosteroid phobia (TOPICOP) scale.

**Materials and methods:**

The TOPICOP scale comprises 12 items grouped into 2 dimensions: beliefs (6 items) and worries (6 items). For cultural adaptation, the scale was forward and backward translated. The final Turkish version was administered to 123 patients with chronic dermatoses who rated their perceptions of TCSs using a 4-point Likert scale. Test-retest reliability was assessed in 30 patients over a 2-week interval. In the validity analysis of the scale, content, construct (exploratory factor analysis (EFA), confirmatory factor analysis (CFA)) and criterion validity were tested. Internal consistency (Cronbach’s alpha coefficient) and test-retest analyses were performed in the reliability analysis.

**Results:**

EFA yielded 2 subdomains explaining 67.61% of the total variance and factor loadings ranging between 0.81 and 0.42. In CFA, χ^2^/df, SRMR, RMSEA, CFI, and TLI were in the excellent/acceptable range. Internal consistency had Cronbach’s alpha coefficients of 0.788 for the beliefs dimension, 0.815 for the worries dimension, and 0.861 for the total scale. A moderate-to-strong positive correlation was observed in test–retest reliability, with a correlation coefficient of 0.746 (p < 0.001). The mean (SD) global score of the Turkish TOPICOP scale was 50.74% (21.06%). Patients with higher educational levels had higher mean scores (p ≤ 0.05).

**Conclusion:**

The Turkish version of the TOPICOP scale is a valid and reliable tool for assessing topical corticophobia among Turkish patients. Physicians should dedicate sufficient time to patient education to enhance TCS adherence.

## 1. Introduction

Topical corticosteroids (TCSs) are the most frequently used drugs in dermatologic practice. Since their introduction over 70 years ago, TCSs have become one of the most important cornerstone treatment options for several inflammatory dermatoses [1,2]. These agents exert their effects by binding to intracellular receptors, regulating the transcription of genes responsible for proinflammatory cytokines. This mechanism underpins their antiinflammatory, antiproliferative, immunosuppressive, and vasoconstrictive properties [3]. However, TCS use is associated with common local side effects, such as skin thinning, purpura, striae, telangiectasia, pigmentation disorders, and acne. Many patients also fear systemic side effects, including weight gain, asthma, growth stunting, and cataracts [4]. Despite these concerns, careful consideration of the potency of an agent and precise adherence to prescribed application frequencies can minimize the risk of adverse effects [5].

The term steroid phobia was first described in 1987 within the context of asthma and eczema [6]. This phenomenon, now more accurately referred to as corticophobia, is prevalent worldwide, with reported rates ranging from 31.0% to 95.7%. Corticophobia often originates from a lack of education regarding treatment, misinformation from nonmedical sources, negative experiences with topical steroids, and fears of adverse effects. Patients with corticophobia tend not to adhere to treatment, which may result in later initiation or earlier discontinuation of prescribed therapies. Nonadherence can result in suboptimal disease control, more hospital visits, and increased healthcare costs [4].

In 2013, the topical corticosteroid phobia (TOPICOP) scale was developed as a standardized tool to assess TCS phobia, particularly in patients with atopic dermatitis [7]. A subsequent international study in 2017 tested the feasibility and comprehensibility of the TOPICOP scale in 17 countries [8]. The TOPICOP scale was originally developed in France and eventually utilized in several countries, including Japan [9], Thailand [10], and Croatia [11]. This study aimed to translate the English version of the TOPICOP scale into Turkish and evaluate its validity and reliability among Turkish patients.

## 2. Materials and methods

This methodological, cross-sectional study was conducted at the Dermatology Outpatient Clinic of Eskişehir Osmangazi University Faculty of Medicine in 2023. The study included 123 adult patients who had been using TCSs for 2–3 months. All patients were asked to fill out an informed consent form before the scale. Exclusion criteria included individuals younger than 18 years old, those with short-term (2–3 months) TCS use for acute dermatological conditions, and those unwilling to participate in the study. Permission to translate and use the TOPICOP scale was obtained from Dr Sébastien Barbarot, the copyright holder, and the study protocol was approved by the local ethics committee (approval number: 48, date: 21 February 2023).

### 2.1. Study population

The study population consisted of patients who applied to the dermatology clinic and used TCSs. The study sample size was based on the principle that validity and reliability studies require 5–10 participants per scale item [12]. Given that the TOPICOP scale consists of 12 items, a total of 123 patients were included. Additionally, test-retest reliability was assessed in 30 patients who agreed to participate in a follow-up evaluation 2 weeks later.

### 2.2. Translation and adaptation process

The TOPICOP scale, originally developed by Moret et al. [7] consists of 12 items divided into 2 dimensions: beliefs (6 items) and worries (6 items). Each item is rated on a 4-point Likert scale, with responses ranging from 0 (totally disagree or never) to 3 (totally agree or always). The total score ranges from 0 to 36, expressed as a percentage, with higher scores indicating more severe corticophobia [7,8].

For the Turkish adaptation, the scale was translated into Turkish by 2 independent language experts. The Turkish version was then back-translated into English by another expert to ensure semantic equivalence. Expert opinions were obtained to finalize the Turkish version, focusing on linguistic and cultural appropriateness.

### 2.3. Data collection

In the first part of the questionnaire, sociodemographic characteristics of the participants such as age, sex, and occupation, as well as information on dermatological diseases and TCS use were recorded. The second part featured the Turkish version of the TOPICOP scale. The third part included the Turkish versions of the Depression Anxiety Stress Scale-21 (DASS-21) and the visual analog scale (VAS) to assess convergent validity. VAS is a single-item measurement commonly used in healthcare settings to measure anxiety related to medical treatments. Patients answered the following question: “If you had to rank your level of fear about using TCS, between no fear and highest fear, where would you place the cursor?” on a VAS of 0–10 to evaluate topical corticophobia. VAS measured corticophobia using a single-item scale ranging from 0 (no fear) to 10 (highest fear). The DASS-21 evaluated depression, anxiety, and stress, with scores ranging from 0 to 3 for each item [13]. The Turkish version of DASS-21 had been validated previously by Yılmaz et al. [14].

### 2.4. The validity and reliability tests of the TOPICOP scale

Regarding construct validity, the suitability of the sample was calculated using Kaiser–Meyer–Olkin (KMO) and Bartlett test values, and the statistical analysis consisted of reliability and validity analyses. Reliability analysis included internal consistency (Cronbach’s alpha) and item-total correlation.

The validity of the Turkish version of TOPICOP was tested with construct validity analysis. Construct validity was assessed using confirmatory factor analysis (CFA) and exploratory factor analysis (EFA). As a result of CFA, acceptable limits of the indexes accepted for goodness-of-fit indicators were as follows: χ^2^/df value below 5 were acceptable, while below 3 indicates very good fit. CFI and NNFI values above 0.90 were sufficient, and above 0.95 indicate very good fit. RMSEA and SRMR values below 0.05 indicated excellent fit, and values below 0.08 indicated good fit [12]. Convergent validity was tested by comparing TOPICOP scores with VAS and DASS-21 scores.

### 2.5. Data analysis

Data analyses were performed using SPSS version 15, Jamovi, and JASP statistical programs. Normality of distribution was assessed using the Kolmogorov–Smirnov test and descriptive statistics. The TOPICOP scale total scores were normally distributed. The DASS-21 scale scores were not normally distributed. In the comparison between groups, t test, ANOVA or Mann–Whitney U, and Kruskal–Wallis tests were used in univariate analyses according to the normal distribution results. Spearman correlation analysis was performed to determine the correlation between the scores of the measurement tools used in the study. All p-values less than 0.05 were considered statistically significant.

## 3. Results

### 3.1. Sociodemographics and clinical characteristics of the sample

The present study recruited 123 patients, with a mean (SD) age of 46.02 years (14.26) (range: 17–84). There were 67 male patients (54.5%), and 56 (45.5%) were female. There were 90 (73.2%) patients that were married and 45 (36.2%) were university graduates. Additionally, 64 (52.0%) of the patients did not have an income-generating job.

There were 108 (87.8%) patients with a history of chronic disease other than dermatological disease, and 33 (26.8%) had a family history of a dermatological disease. Regarding the use of TCSs, 86 (69.9%) were being treated for psoriasis, and 19 (15.4%) for dermatitis, including contact dermatitis (n = 9), atopic dermatitis (n = 5), lichenoid dermatitis (n = 4), and seborrheic dermatitis (n = 1). Of the remaining patients, 7 (5.6%) were diagnosed with mycosis fungoides, 7 (5.6%) with autoimmune bullous diseases, 2 (1.6%) with vitiligo, and 1 (0.8%) each with subacute cutaneous lupus erythematosus and morphea. Among the patients, 110 (89.4%) had a disease duration of 1 year or more, and 89 (72.4%) had been using TCS for over a year. Regarding usage frequency, 48 (39.0%) applied TCS once daily, 45 (36.6%) twice daily, 22 (17.9%) only on weekends, and 8 (6.5%) 3 times a day.

### 3.2. Knowledge level regarding TCSs

Of the patients, 113 (91.9%) were knowledgeable about proper TCS usage and 101 (82.1%) had received this information from a physician. However, only 45 (36.6%) were aware of the potential side effects of TCSs. The most commonly known side effect was thinning of the skin (19.5%). Other reported side effects included weight gain (5.7%), sensitivity (4.9%), burning (2.4%), excessive hair growth (2.4%), increased lesions (1.6%), edema (1.6%), liver damage (1.6%), and one case each of redness, bleeding, immune system problems, dryness, itching, or elevated blood sugar levels (0.8%).

### 3.3. Validity and reliability analysis of the Turkish version of TOPICOP scale

Cronbach’s alpha coefficients were 0.788 for the beliefs dimension, 0.815 for the worries dimension, and 0.861 for the total scale. When any item was removed, Cronbach’s alpha varied between 0.843 and 0.858. The item-total correlation coefficients ranged from 0.421 to 0.661. The CFA showed that all fit indices were within acceptable ranges (Table 1). The path diagram of the confirmatory factor analysis is presented in the Figure.

A test–retest analysis was performed with 30 patients over a 2-week interval. The mean (SD) pretest scores for the beliefs, worries, and total dimensions were 8.20 (2.95) (45.5% (16.3%)), 9.40 (4.60) (52.2% (25.5%)), and 17.60 (7.0) (48.4% (19.4%)), respectively. The retest scores for the same dimensions were 5.47 (2.97) (30.3% (16.5%)), 6.47 (3.39) (35.9% (18.8%)), and 11.93 (5.49) (33.1% (15.2%)), respectively. The correlation coefficient between total test and retest scores was 0.746 (p < 0.001), indicating a moderate-to-strong positive correlation.

### 3.4. The Turkish version of the TOPICOP scale

The mean (SD) TOPICOP scale score was 8.31 (4.17) (46.16% (23.13%)) for the beliefs dimension, 9.96 (4.42) (55.32% (24.53%)) for the worries dimension, and 18.27 (7.58) (50.74% (21.06%)) for the total score. When questioned about their beliefs and worries regarding TCSs, 50 (40.7%) of the patients indicated that “I am afraid of putting TCS on certain skin areas such as eyelids”, while 39 (31.7%) of the patients reported that “I need reassurance about TCS.” Additionally, 36 (29.3%) of the patients reported that “I stop TCS treatment as soon as I can.” Patients’ responses to each item of the TOPICOP scale are presented in Table 2.

The mean values of the 2 dimensions and total were evaluated in relation to sociodemographic features. No statistically significant differences were found in terms of geriatric patients, sex, marital status, presence of chronic disease, TCS usage period, and having information about TCS. However, the mean TOPICOP “worries” dimension score and mean “global” score of TOPICOP scale were statistically lower in those with less than 8 years of education than in those with more than 8 years of education (p ≤ 0.05) (Table 3).

### 3.5. Correlation between VAS and DASS-21 with TOPICOP scale scores

A statistically significant positive correlation was found between both the median scores of beliefs and worries dimensions of TOPICOP scale and the median VAS score (p < 0.001). Furthermore, the median total TOPICOP scale score was positively correlated with the median VAS score and the anxiety and stress subscales of DASS-21 (p ≤ 0.05) (Table 4).

## 4. Discussion

The present study adapted the TOPICOP scale into Turkish and assessed criterion-related validity. The Turkish version of the scale, comprising 12 items, showed internal consistency across both dimensions and the overall score. The CFA further confirmed that all fit indices were within acceptable ranges. Similar to previous cultural adaptations [9–11], our findings indicate that the Turkish version of the TOPICOP scale is both valid and reliable.

Based on the application of the scale to the same individuals twice over a 2-week interval, a significant decrease was observed in both the total TOPICOP scale and the scores in its subdimensions at the end of the 14th day. This may be due to the awareness regarding the implementation of the survey, the effect of the education given to the patients by clinicians, and/or the decrease in concerns related to the treatment.

In our study, more than 80% of the patients were aware of proper TCS usage, with physicians being the primary source of this information. Additionally, thinning of the skin was the most commonly known side effect among the patients. Consistent with our findings, Alamri et al. [15] reported that physicians were the main source of information for patients with dermatological conditions regarding TCSs. Conversely, a South Korean study found that over half of the participants were unaware of TCSs side effects [16], while a Saudi cross-sectional study found that 61.3% of participants were unaware of the risks associated with TCSs misuse on the face [17]. This is of great importance since physicians play a crucial role in informing and treating patients.

In a large international feasibility study of the TOPICOP scale, including both children (80.1%) and adult (19.9%) atopic dermatitis patients, the highest scores were reported in Poland (58.4%), Ukraine (55.1%), and Taiwan (52.9%), and the lowest in Germany (38.5%), Denmark (35.4%), and Brazil (33.5%) [8]. Christensen et al. [18] evaluated the corticosteroid phobia in patients with chronic hand eczema and the median TOPICOP scale score was 51.9 ± 23.2% in their study. Another study on dermatology patients reported a median global TOPICOP score of 44.4 ± 17.6% [19]. The findings of our study found that the mean (SD) global TOPICOP scale score was 18.27 (7.58) (50.74% (21.0%)). Although our mean global TOPICOP scale scores were higher compared to studies in the literature [9,19–22], our results were similar with the study of Christensen et al. [18]. These variations in global TOPICOP scores may be related to both differences in study populations and cultural beliefs.

According to the Turkish version of the TOPICOP scale, 50 (40.7%) patients expressed fear of applying TCSs to specific areas, such as the eyelids, and 39 (31.7%) reported needing reassurance about TCSs usage. These concerns were similarly prevalent in the Thai adaptation of the scale [10] and have been widely documented in the literature [18,19]. Such apprehensions may have arisen from physicians cautioning patients about applying TCSs to sensitive areas like the eyes.

It is documented that populations more likely to be reluctant to use TCSs included female patients, those who frequently changed dermatology providers, and those who received conflicting information from healthcare providers [19,20,26,27]. Additionally, some studies reported a correlation between age and topical corticophobia, specifically in younger patients [28] and patients less than 60 years of age [27]; while other studies have found no connection [19,29,30]. Our study found no relationship between the patients’ sociodemographic characteristics such as age, sex, and marital status and TOPICOP scores.

Today, swift access to information and the proliferation of conspiracy theories about side effects have contributed to the increased anxiety and concerns about the use of TCSs in many patients [23,24]. Patients may reject TCSs due to misinformation or inadequate education regarding the treatment [23,25]. It is well known that topical corticophobia is inversely proportional to the level of health education of patients [4]. Conversely, increased health literacy and easy access to online resources might be associated with higher corticophobia rather than the educational status of the person themselves [31]. In our study, we could not evaluate the health literacy of the patients. However, patients with more than 8 years of education had statistically higher corticophobia. This indicates that even highly educated patients may have difficulties in selecting correct and trustworthy medical information from the media. Therefore, clinicians should provide more educational interventions to improve patients’ confidence in using TCSs.

In our study, we also used the VAS as a screening tool to assess topical corticophobia. Higher VAS scores were positively correlated with higher TOPICOP scores. Additionally, we used the DASS-21 as the parallel test of the Turkish version of TOPICOP scale to assess the patients’ mental status to show that anxious and stressed individuals were more likely to have corticophobia.

The main limitation of our study is the predominance of psoriasis patients in our sample that may affect the generalizability of our findings. While the TOPICOP scale was initially designed for patients with atopic dermatitis, it has been widely used to measure corticophobia in other chronic dermatological conditions, including psoriasis [21,27,32,33]. Although TCS concerns have not been specifically studied in psoriasis patients, it is well known that fear of side effects is a key reason for poor adherence to TCS treatment [34]. Additionally, psoriasis patients have comparable TOPICOP scores to those with atopic dermatitis, indicating the applicability of the scale to psoriasis patients (32). Our study is the first nationwide effort to assess corticophobia in Turkish patients with chronic dermatological conditions using a validated and detailed questionnaire. Given the global variability of the prevalence of diseases, we believe the TOPICOP scale is applicable to patients with chronic dermatological conditions using TCSs.

In conclusion, the Turkish version of the TOPICOP scale is a valid and reliable tool for assessing topical corticophobia among Turkish patients. Our findings align with other validation studies, highlighting the psychometric robustness of the scale. Enhancing patient education may help alleviate concerns about TCS use, improving adherence and treatment outcomes. Healthcare providers and dermatologists should allocate time to address patients’ concerns to optimize therapy. The Turkish TOPICOP scale offers a practical framework for future interventional studies aimed at improving long-term adherence to TCSs.

## Supplementary Information

**Supplement 1:** Turkish version of the TOPICOP scale.

		Kesinlikle katılmıyorum	Pek Katılmıyorum	Kısmen katılıyorum	Kesinlikle katılıyorum
1.	Topikal kortikosteroidler kan dolaşımına geçer.				
2.	Topikal kortikosteroidler enfeksiyonlara yol açabilir.				
3.	Topikal kortikosteroidler kilo aldırır.				
4.	Topikal kortikosteroidler cildinize zarar verir.				
5.	Topikal kortikosteroidler gelecekte sağlığımı etkileyecektir.				
6.	Topikal kortikosteroidler astıma yol açabilir.				
7.	Topikal kortikosteroidlerin herhangi bir yan etkisini bilmiyorum ama yine de korkuyorum.				
		Hiçbir zaman	Bazen	Sıklıkla	Her zaman
8.	Çok fazla krem sürmekten korkuyorum.				
9.	Göz kapakları gibi cildin daha ince olduğu belirli bölgelere krem sürmekten korkuyorum.				
10.	Topikal kortikosteroid kullanmaya başlamadan önce bekleyebildiğim kadar beklerim.				
11.	Topikal kortikosteroid tedavisini mümkün olan en kısa sürede sonlandırırım.				
12.	Topikal kortikosteroidler konusunda güvenceye ihtiyacım var.				

## Figures and Tables

**Figure:**
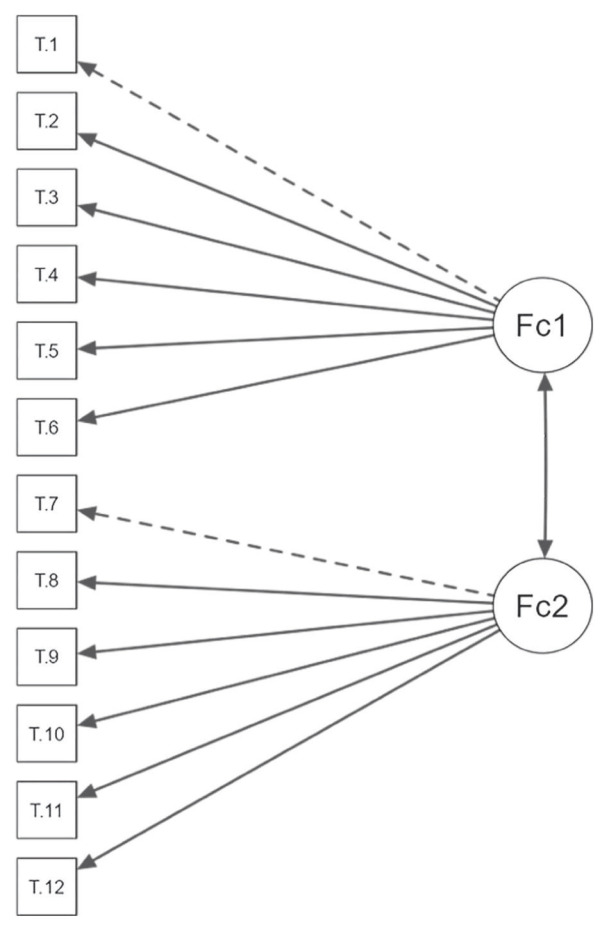
Path diagram.

**Table 1:** Validity analysis of the TOPICOP scale.

	Mean (SD)	Item-rest correlation	If item dropped Cronbach’s alfa	Factor loadings
**Beliefs dimension**				
**1**. TCS pass into the bloodstream.	1.43 (0.91)	0.494	0.854	0.518
**2**. TCS can lead to infections.	1.32 (0.84)	0.421	0.858	0.421
**3**. TCS make you fat.	1.37 (1.11)	0.541	0.851	0.753
**4**. TCS damage your skin.	1.63 (1.00)	0.582	0.848	0.711
**5**. TCS will affect my health in the future.	1.51 (1.04)	0.661	0.843	0.815
**6**. TCS can lead to asthma.	1.05 (0.89)	0.535	0.852	0.509
**Cronbach’s alpha value: 0.788** **Variance%: 52.26%**				
**Worries dimension**				
**7**. I do not know of any side effects of TCS, but I’m still afraid.	1.36 (1.03)	0.505	0.854	0.518
**8**. I am afraid of applying too much cream.	1.61(1.04)	0.585	0.848	0.662
**9**. I am afraid of putting cream on certain areas like my eyelids, where the skin is thinner.	1.99 (1.02)	0.472	0.856	0.652
**10**. I wait as long as I can before treating myself with TCS.	1.51 (1.04)	0.548	0.851	0.691
**11**. I stop the TCS treatment as soon as I can.	1.72 (1.05)	0.544	0.851	0.686
**12**. I need reassurance about TCS.	1.76 (1.03)	0.588	0.848	0.757
**Cronbach’s alpha value: 0.815;** **Variance%: 15.34%**				
**Total scale: Cronbach’s alpha value: 0.861; variance%: 67.61%** **KMO: 0.860; Bartlett’s test: 504,270; p < 0.001** **χ2/df = 1.68; CFI: 0.922; TLI: 0.903; SRMR: 0.0658; RMSEA: 0.0746**				

**Table 2:** Distribution of responses to TOPICOP scale items.

Items		Totally disagree/never n (%)	Not really agree/sometimes n (%)	Almost agree/often n (%)	Totally agree/always n (%)
**1**.	TCS pass into the bloodstream.	20 (16.3)	45 (36.6)	43 (35.0)	15 (12.2)
**2**.	TCS can lead to infections.	20 (16.3)	54 (43.9)	39 (31.7)	10 (8.1)
**3**.	TCS make you fat.	35 (28.5)	33 (26.8)	29 (23.6)	26 (21.1)
**4**.	TCS damage your skin.	20 (16.3)	33 (26.8)	43 (35.0)	27 (22.0)
**5**.	TCS will affect my health in the future.	24 (19.5)	39 (31.7)	33 (26.8)	27 (22.0)
**6**.	TCS can lead to asthma.	37 (30.1)	51 (41.5)	27 (22.0)	8 (6.5)
**7**.	I do not know of any side effects of TCS, but I’m still afraid.	31 (25.2)	37 (30.1)	35 (28.5)	20 (16.3)
**8**.	I am afraid of applying too much cream.	22 (17.9)	33 (26.8)	39 (31.7)	29 (23.6)
**9**.	I am afraid of putting cream on certain areas like my eyelids, where the skin is thinner.	13 (10.6)	25 (20.3)	35 (28.5)	50 (40.7)
**10**.	I wait as long as I can before treating myself with TCS.	23 (18.7)	41 (33.3)	32 (26.0)	27 (22.0)
**11**.	I stop the TCS treatment as soon as I can.	19 (15.4)	32 (26.0)	36 (29.3)	36 (29.3)
**12**.	I need reassurance about TCS.	15 (12.2)	38 (30.9)	31 (25.2)	39 (31.7)

**Table 3:** Comparison of TOPICOP scale scores of the participants according to the sociodemographic and clinic variables.

	TOPICOP scale			

Variables n =123	Dimensions		Total (T) score Mean (SD)	p

	Beliefs (B) Mean (SD)	Worries (W) Mean (SD)		

**Age groups**				W:0.084
<65 years	8.52 (4.04)	10.08 (4.74)	18.60 (7.47)	B:0.355
≥ 65 years	6.33 (4.96)	8.83 (3.81)	15.17 (8.24)	T:0.136

**Sex n(%)**				W:0.220
Male	8.73 (4.08)	10.18 (4.33)	18.91 (7.60)	B:0.548
Female	7.80 (4.25)	9.70 (4.54)	17.50 (7.56)	T:0.306

**Educational level**				**W:0.040**
≤8 years	7.20 (4.41)	9.05 (4.16)	16.25 (7.49)	B:0.113
>8 years	8.84 (3.60)	10.40 (4.49)	19.24 (7.48)	**T:0.040**

**Marital status n (%)**				W:0.586
Married	8.43 (4.12)	9.69 (4.42)	18.12 (7.57)	B:0.264
Single	7.97 (4.33)	10.70 (4.38)	18.67 (7.72)	T:0.726

**Chronic disease**				W:0.675
Present	8.73 (4.13)	11.47 (4.34)	20.20 (7.51)	B:0.159
Absent	8.25 (4.18)	9.75 (4.41)	18.00 (7.59)	T:0.294

**Systemic medications**				W:0.148
Present	8.89 (3.79)	10.51 (4.38)	19.40 (7.08)	B:0.201
Absent	7.80 (4.43)	9.48 (4.42)	17.29 (7.91)	T:0.123

**TCS usage period**				W:0.597
<12 months	8.69 (2.46)	9.38 (3.15)	18.08 (4.35)	B: 0.622
≥12 months	8.26 (4.33)	10.03 (4.55)	18.29 (7.89)	T:0.882

**Getting information about TCS**				W:0.699
Yes	8.27 (4.16)	9.90 (4.46)	18.17 (7.34)	B:0.634
No	8.80 (4.47)	10.60 (4.03)	19.40 (5.74)	T:0.624

* TCS: topical corticosteroids, SD: standard deviation.

**Table 4:** Correlation analysis of TOPICOP scale scores with VAS and DASS-21 scores.

Variables		TOPICOP scale		

		Beliefs dimension r; p	Worries dimension r; p	Total score r; p

**VAS**				
Median (min–max)	5.0 (0–10)	0.406; **<0.001**	0.330; **<0.001**	0.414; **<0.001**

**DASS-21 Depression subscale**				
Median (min–max)	3.0 (0–15)	0.107; 0.237	0.215; **0.017**	0.168; 0.063

**DASS-21 Anxiety subscale**				
Median (min–max)	3.0 (0–16)	0.192; **0.033**	0.204; **0.024**	0.212; **0.019**

**DASS-21 Stress subscale**				
Median (min–max)	5.0 (0–15)	0.127;0.162	0.247; **0.006**	0.207; **0.022**

## References

[1] Hengge UR Ruzicka T Schwartz RA Cork MJ Adverse effects of topical glucocorticosteroids Journal of the American Academy of Dermatology 2006 54 1 1 15 10.1016/j.jaad.2005.01.010 16384751

[2] Brazzini B Pimpinelli N New and established topical corticosteroids in dermatology: clinical pharmacology and therapeutic use American Journal of Clinical Dermatology 2002 3 1 47 58 10.2165/00128071-200203010-00005 11817968

[3] Schadt CR Jackson SM Glucocorticosteroids Bolognia JL Schaffer JV Cerroni L Dermatology 5th ed Poland Elsevier Saunders 2025 2217 2230

[4] Contento M Cline A Russo M Steroid Phobia: a review of prevalence, risk factors, and interventions American Journal of Clinical Dermatology 2021 22 6 837 851 10.1007/s40257-021-00623-6 34287768

[5] Mehta AB Nadkarni NJ Patil SP Godse KV Gautam M Topical corticosteroids in dermatology Indian Journal of Dermatology, Venereology and Leprology 2016 82 4 371 378 10.4103/0378-6323.178903 27279294

[6] David TJ Steroid scare Archives of Disease in Childhood 1987 62 9 876 878 10.1136/adc.62.9.876 3674940 PMC1778574

[7] Moret L Anthoine E Aubert-Wastiaux H Le Rhun A Leux C TOPICOP©: a new scale evaluating topical corticosteroid phobia among atopic dermatitis outpatients and their parents PLoS One 2013 8 10 e76493 10.1371/journal.pone.0076493 24146878 PMC3797828

[8] Stalder JF Aubert H Anthoine E Futamura M Marcoux D Topical corticosteroid phobia in atopic dermatitis: international feasibility study of the TOPICOP score Allergy 2017 72 11 1713 1719 10.1111/all.13189 28439896

[9] Futamura M Yamamoto-Hanada K Saito M Batchelor J Nakahara M The Japanese version of Topicop scale among patients with atopic dermatitis: a translation and feasibility study Arerugi 2016 65 1 66 72 10.15036/arerugi.65.66 26923656

[10] Boonpuen N Srimuang A Puangpet P Supsrisunjai C Validity and reliability of the topical corticosteroid phobia (TOPICOP©) questionnaire: Thai version Siriraj Medical Journal 2023 75 2 115 120 10.33192/smj.v75i2.260750

[11] Markota Čagalj A Markic J Vuković D Šitum Čeprnja Z Gogić Salapić T Linguistic validation and reliability of the Croatian version of the TOPICOP questionnaire Medicina (Kaunas) 2024 60 6 968 10.3390/medicina60060968 38929585 PMC11205604

[12] Schreiber JB Nora A Stage FK Barlow EA King J Reporting structural equation modeling and confirmatory factor analysis results: a review The Journal of Educational Research 2006 99 6 323 338 10.3200/JOER.99.6.323-338

[13] Lovibond PF Lovibond SH The structure of negative emotional states: comparison of the Depression Anxiety Stress Scales (DASS) with the Beck Depression and Anxiety Inventories Behaviour Research and Therapy 1995 33 3 335 343 10.1016/0005-7967()00075-U94 7726811

[14] Yılmaz Ö Boz H Arslan A Depresyon Anksiyete Stres Ölçeğinin (DASS 21) Türkçe kısa formunun geçerlilik-güvenilirlik çalışması Finans Ekonomi ve Sosyal Araştırmalar Dergisi (FESA) 2017 2 2 78 91 (in Turkish with an abstract in English).

[15] Alamri RA Al Satti HS Knowledge and attitudes towards topical corticosteroids among previous users in the general population of Saudi Arabia Cureus 2024 16 3 e55373 10.7759/cureus.55373 38562369 PMC10983776

[16] Seo H Song SY Kim D Park JH Shin Y General public knowledge regarding topical corticosteroids: a nationwide survey in South Korea Korean Journal of Clinical Pharmacy 2022 32 2 84 92 10.24304/kjcp.2022.32.2.84

[17] Al Dhafiri M Alali AB Alghanem ZA Alsaleh ZW Boushel EA Topical steroid damaged face: a cross-sectional study from Saudi Arabia Clinics and Practice 2022 12 1 140 146 10.3390/clinpract12010018 35200269 PMC8870366

[18] Christensen MO Sieborg J Nymand LK Guttman-Yassky E Ezzedine K Prevalence and clinical impact of topical corticosteroid phobia among patients with chronic hand eczema-Findings from the Danish Skin Cohort Journal of the American Academy of Dermatology 2024 91 6 1094 1103 10.1016/j.jaad.2024.07.1503 39181406

[19] Choi E Chandran NS Tan C Corticosteroid phobia: a questionnaire study using TOPICOP score Singapore Medical Journal 2020 61 3 149 153 10.11622/smedj.2019110 32488277 PMC7905116

[20] Gerner T Haugaard JH Vestergaard C Deleuran M Jemec GB Healthcare utilization in Danish children with atopic dermatitis and parental topical corticosteroid phobia Pediatric Allergy and Immunology 2021 32 2 331 341 10.1111/pai.13394 33047404

[21] Starbek Zorko M Benko M Rakuša M Prunk Zdravković T Evaluation of corticophobia in patients with atopic dermatitis and psoriasis using the TOPICOP© score Acta Dermatovenerologica Alpina, Pannonica, et Adriatica 2023 32 4 135 139 10.15570/actaapa.2023.26 38126095

[22] Saito-Abe M Futamura M Yamamoto-Hanada K Yang L Suzuki K Topical corticosteroid phobia among caretakers of children with atopic dermatitis: a cross-sectional study using TOPICOP in Japan Pediatric Dermatology 2019 36 3 311 316 10.1111/pde.13784 30882946

[23] Finnegan P Murphy M O’Connor C #corticophobia: a review on online misinformation related to topical steroids Clinical and Experimental Dermatology 2023 48 2 112 115 10.1093/ced/llac019 36730502

[24] Lambrechts L Gilissen L Morren MA Topical corticosteroid phobia among healthcare professionals using the TOPICOP score Acta Dermato-Venereologica 2019 99 11 1004 1008 10.2340/00015555-3220 31099401

[25] Gomes TF Kieselova K Guiote V Henrique M Santiago F A low level of health literacy is a predictor of corticophobia in atopic dermatitis Anais Brasileiros de Dermatologia 2022 97 704 709 10.1016/j.abd.2021.11.007 36057460 PMC9582876

[26] Kojima R Fujiwara T Matsuda A Narita M Matsubara O Factors associated with steroid phobia in caregivers of children with atopic dermatitis Pediatric Dermatology 2013 30 1 29 35 10.1111/j.1525-1470.2012.01808.x 22747965

[27] Mueller SM Itin P Vogt DR Walter M Lang U Assessment of “corticophobia” as an indicator of non-adherence to topical corticosteroids: a pilot study Journal of Dermatological Treatment 2017 28 2 104 111 10.1080/09546634.2016.1201189 27396480

[28] Song SY Jung SY Kim E Steroid phobia among general users of topical steroids: a cross-sectional nationwide survey Journal of Dermatological Treatment 2019 30 3 245 250 10.1080/09546634.2018.1508817 30081700

[29] Bos B Antonescu I Osinga H Veenje S de Jong K Corticosteroid phobia (corticophobia) in parents of young children with atopic dermatitis and their health care providers Pediatric Dermatology 2019 36 1 100 104 10.1111/pde.13698 30338542

[30] Raffin D Giraudeau B Samimi M Machet L Pourrat X Corticosteroid phobia among pharmacists regarding atopic dermatitis in children: a national French survey Acta Dermato-Venereologica 2016 96 2 177 180 10.2340/00015555-2157 26039683

[31] Dufresne H Bataille P Bellon N Compain S Deladriere E Risk factors for corticophobia in atopic dermatitis Journal of European Academy of Dermatology and Venereology 2020 34 12 e846 9 10.1111/jdv.16739 32526095

[32] Moawad S Mahé E Aubert-Wastiaux H Phan A Maruani A Topical corticosteroid concerns among parents of children with psoriasis versus atopic dermatitis: a French multicenter cross-sectional study American Journal of Clinical Dermatology 2018 19 261 265 10.1007/s40257-017-0318-5 28849428

[33] Choi E Tan KW Tang F Tan C Chandran NS Efficacy of targeted education in reducing topical steroid phobia: a randomized clinical trial Journal of the American Academy of Dermatology 2020 83 6 1681 1687 10.1016/j.jaad.2020.02.079 32171815

[34] Brown KK Rehmus WE Kimball AB Determining the relative importance of patient motivations for nonadherence to topical corticosteroid therapy in psoriasis Journal of the American Academy of Dermatology 2006 55 4 607 613 10.1016/j.jaad.2005.12.021 17010739

